# HLA allele-specific expression: Methods, disease associations, and relevance in hematopoietic stem cell transplantation

**DOI:** 10.3389/fimmu.2022.1007425

**Published:** 2022-09-28

**Authors:** Tiira Johansson, Jukka Partanen, Päivi Saavalainen

**Affiliations:** ^1^ Translational Immunology Research Program, Research Programs Unit, University of Helsinki, Helsinki, Finland; ^2^ Research and Development, Finnish Red Cross Blood Service, Helsinki, Finland; ^3^ Genetics Research Program, Folkhälsan Research Center, Helsinki, Finland

**Keywords:** human leucocyte antigen, next generation sequencing, RNA sequencing, allele-specific expression, disease associations

## Abstract

Varying HLA allele-specific expression levels are associated with human diseases, such as graft versus host disease (GvHD) in hematopoietic stem cell transplantation (HSCT), cytotoxic T cell response and viral load in HIV infection, and the risk of Crohn’s disease. Only recently, RNA-based next generation sequencing (NGS) methodologies with accompanying bioinformatics tools have emerged to quantify HLA allele-specific expression replacing the quantitative PCR (qPCR) -based methods. These novel NGS approaches enable the systematic analysis of the HLA allele-specific expression changes between individuals and between normal and disease phenotypes. Additionally, analyzing HLA allele-specific expression and allele-specific expression loss provide important information for predicting efficacies of novel immune cell therapies. Here, we review available RNA sequencing-based approaches and computational tools for NGS to quantify HLA allele-specific expression. Moreover, we explore recent studies reporting disease associations with differential HLA expression. Finally, we discuss the role of allele-specific expression in HSCT and how considering the expression quantification in recipient-donor matching could improve the outcome of HSCT.

## Introduction

Due to their biological role of presenting peptide antigens to T cells, the highly polymorphic HLA class I and class II molecules are crucial for T cell activation and therefore for effective immune response against various pathogens, autoantigens, alloantigens, and cancer (1, 2). HLA class I molecules, which are constitutively expressed on the surface of nearly all nucleated cells present intracellular peptides to CD8^+^ T cells and are encoded by genes *HLA-A*, *-B*, and *-C* (3). In contrast, HLA class II molecules, which are expressed on professional antigen presenting cells (APCs) such as B cells, macrophages, and dendritic cells (DCs) display extracellular peptides to CD4^+^ T cells and are encoded by *HLA-DRA*, *-DRB1-9*, *-DQA1*, *-DQB1*, *-DPA1*, and *-DPB1* (4). In addition to the classical HLA, the low-polymorphic non-classical HLA molecules such as *HLA-E*, *-F*, and *-G* and *HLA-DM* and *-DO* have important roles in immunosuppression (5, 6) and peptide loading (7). Several factors affect HLA expression (**Table 1**). For example, differential expression levels have been demonstrated between different HLA genes, alleles, tissues, and cell types (8, 9, 12, 13, 15, 30). Part of this variation emerges from structural differences in the promoter motifs involved in transcriptional expression regulation between HLA genes and alleles (31, 32). Besides, multiple transcriptional and translational factors as well as proinflammatory cytokines can affect the mRNA- and surface-level expression of HLA (18, 31, 33, 34). Varying expression may also result from individual-specific factors such as genetic polymorphism, age, environment, and medication (25–28). Since many of these factors affecting HLA expression have been previously described in detail e.g. by Carey et al. (18) and Petersdorf et al. (19), in this mini review, we will focus more on the RNA-based methods in HLA allele-specific expression quantification and known disease associations with HLA expression.

**Table 1:** Factors associated with differential HLA expression levels.

Factor	Effect on expression	References
Gene	Expression levels can vary between different HLA genes e.g. HLA class I genes are expressed at higher levels than class II genes.	(8–11)
Allele	Expression can vary between HLA alleles due to genetic polymorphisms.	(8, 9, 11–14)
Tissue and cell	HLA expression and turnover rates of HLA molecules can be tissue- and cell-specific.	(15–17)
Promoter polymorphisms	Proximal and distal promoter polymorphisms have been associated with differential HLA expression.	(12, 18, 19)
Alternative splicing	Alternative splicing may lead to misfolded HLA proteins and aberrant expression.	(18, 20)
Epigenetic regulation	DNA methylation can alter HLA expression e.g. the methylation level in high-expression HLA-A allotypes is higher than in low-expression HLA-A allotypes.	(18)
Turnover and stability of mRNA and protein	Post-transcriptional and -translational factors can affect the degradation and internalization rates of HLA molecules.	(18)
HLA LOH	In cancer, the decrease of HLA expression can be dependent on the different forms of HLA LOH (total or partial) resulting from genetic mutations and chromosomal aberrations.	(21, 22)
Proinflammatory cytokines	Depending on the gene, HLA expression can either be upregulated or downregulated by proinflammatory cytokines.	(23, 24)
Age	Ageing can alter class I and class II expression.	(25, 26)
Medication and environment	Medication and environmental factors such as diet can alter HLA expression.	(27, 28)
Time point	HLA allele-specific expression can vary between different time points in activated memory T cells.	(29)
Cell composition of study sample	HLA expression can vary between different cell types and thus when HLA expression is quantified from bulk RNA-seq, cell composition should be taken into account.	(15)

LOH, loss of heterozygosity.

Over the past decade, studies have associated differential HLA expression levels with infectious and autoimmune diseases, neurological disorders, cancer, and drug hypersensitivity (35–39). Additionally, HLA expression variation has been shown to impact the outcomes of flow cytometric crossmatches and HSCT (10, 40, 41). The earlier studies have applied methods such as flow cytometry, qPCR, and microarray in the quantification of HLA expression at the protein- and mRNA-level (12, 42–45). Although these methods have offered valuable information of HLA expression and its associations with human diseases, they are laborious, time-consuming, and do not allow high-resolution and high-throughput expression quantification. Thus, RNA sequencing (RNA-seq) enabling accurate and massively parallel analysis, provides a powerful tool for studying inter-allelic and inter-individual expression differences in healthy tissues and diseases (46). Since determination of HLA gene- and allele-level expression is important in several applications such as biomarker discovery, transplantation medicine, development of cancer vaccines targeting neo-epitopes, and disease susceptibility (41, 47–49), RNA-seq methods together with novel computational software capable of measuring HLA expression at the allele-level (8, 9, 11, 13, 14, 50) hold great promise to meet this need.

## Methods in HLA expression quantification

### Flow cytometry, qPCR, and microarrays

Different methods exist for HLA expression quantification. At the protein-level, HLA expression at the cell surface can be quantified with fluorolabeled monoclonal antibodies and flow cytometry. Earlier studies have used flow cytometry to compare cell surface expression between different HLA genes as well as to explore the gene-specific changes in constitutive and induced HLA expression (45, 51). Together with a specific antibody flow cytometry has revealed differential *HLA-C* surface expression between *HLA-C* allotypes in healthy individuals (42, 44). Moreover, flow cytometry has been used to demonstrate how sequence variation in the coding and non-coding area and the turnover of heavy chain mRNA can modulate *HLA-C* protein expression (44, 52–54). Before the advent of NGS technologies, the level of HLA transcription was quantified with qPCR and microarrays (12, 43, 54–59). Studies using qPCR have demonstrated the impact of distinct DNA methylation patterns and genomic variants in differential expression between divergent HLA allelic lineages (43, 55). Moreover, at the allele-level, a study with qPCR associated high allele-specific expression variation in *HLA-C* with HLA-extended haplotypes (12). These more conventional methods, however, have certain limitations. They require careful design and selection of antibodies, PCR primers, and microarray probes to equally capture the high allelic variation of HLA (37). Potential biases introduced at this step may lead to specificity and sensitivity issues and ambiguous results in the downstream analyses.

### RNA-based NGS methods for HLA allele-specific expression quantification

Over the past decade, NGS has rapidly replaced the more conventional HLA typing methods providing more accurate high-resolution typing (60, 61). In addition, RNA-based NGS technologies have enabled simultaneous expression analysis and identification of expression quantitative trait loci (eQTLs) (11). Due to this revolution, several RNA-seq methods have emerged allowing accurate HLA allele-specific expression quantification. After a common step of reverse-transcribing RNA into complementary DNA (cDNA), these methods rely on different approaches in the sequencing library preparation such as using whole transcriptome data (9, 10) or enriching HLA genes either with PCR amplification with universal gene-specific primers (9, 13, 62) or capturing them using biotinylated oligonucleotide probes covering the target sequences (8). The first published method was based on cDNA amplicon pyrosequencing using a common universal primer to enrich all class I genes (62). Although, it was initially developed for HLA typing, it was later applied also in a study revealing differential allele-specific expression in human and macaque leukocyte subsets (63). A method capturing and enriching the targeted HLA sequences in a hybridization step prior to sequencing has also been successful in quantifying HLA allele-specific expression (8). The method enabled both HLA genotyping and quantification of HLA allele-specific expression of 12 classical genes from healthy PBMC and umbilical cord bloods samples. By using an in-house method for the Illumina RNA-seq reads, the authors reported differential allele-specific expression in several HLA genes. In our method, we incorporated unique molecular identifiers (UMIs) by using template-switching oligo (TSO) during first-strand synthesis in the library preparation to accurately quantify HLA gene- and allele-specific mRNA expression from PBMC samples of healthy individuals (9). Since UMIs enable the removal of PCR bias in the data analysis step, the expression quantification was based on solely counting the original mRNA transcripts in the sample (64). By quantifying unique UMIs per allele with HLAXPress pipeline, we identified differential expression levels between distinct HLA genes, alleles, and haplotypes. Although, many of the RNA-seq methods in HLA research have used Illumina’s short-read technology, there are also a few studies, which have applied Oxford Nanopore Technology’s (ONT) long-reads in expression quantification (10, 13). A recent study quantified HLA allele-level expression using UMIs from ONT HLA-gene specific PCR amplicons (13). In addition to the high-resolution HLA typing of classical class I and class II genes, the assay provided allele-specific HLA expression quantification using Athlon2 pipeline (65) fast enough to be considered in graft allocation for transplantation from a deceased donor. Moreover, ONT’s whole transcriptome data without any HLA gene enrichment step was demonstrated to be sufficient for accurate HLA typing and measuring of the gene-level expression of HLA class I genes (10). There is also evidence that T cell activation can alter the balance of allele-specific expression (29). Interestingly, by using Illumina’s whole transcriptome data together with an HLA-personalized reference for individuals, the authors showed that the expression balance between two alleles in a heterozygous individual changed over time.

### Computational tools for HLA expression quantification from existing RNA-seq datasets

In addition to laboratory protocols, several computational tools have been developed for RNA-seq data, allowing HLA expression quantification from existing public datasets (11, 14, 50, 66, 67). The obvious advantage here is that large, well-documented study materials may be utilized for detailed HLA expression studies.

Seq2HLA, a python- and R-based in silico -method, was the first tool, which was introduced as capable of quantifying HLA expression from RNA-seq data (50). The tool accepts standard RNA-seq reads in fastq format as an input, uses bowtie (68) in aligning reads against exon 2 and 3 sequences of HLA alleles, and outputs HLA types and class-level expression estimates. When applied to a paired-end data with a read length of 37 bp of previously HLA genotyped individuals, seq2HLA achieved 100% specificity and 93.5% sensitivity in 1-field genotyping results. Since seq2HLA was first introduced, two other studies have applied it to quantify HLA expression in human cancer cell lines and non-cancer human tissues and cell types demonstrating HLA expression variation between cancer types and distinct anatomical sites (15, 16).

AltHapAlignR provides estimates of transcript abundance by using alternate reference haplotypes (14). AltHapAlignR first aligns reads against the standard genome reference using read mappers such as Tophat2 (69), HISAT2 (70), or STAR (71) and then extracts reads mapping to HLA region and unmapped reads, which it further re-aligns to the HLA reference haplotypes to obtain the expression estimates for HLA genes and haplotypes. The authors showed that when compared to the standard single reference mapping, AltHapAlignR improved the accuracy of HLA expression quantification. Recently, AlthHapAlignR was applied in a study demonstrating allele-specific expression in *HLA-DRB1* in patients with rheumatoid arthritis and in healthy controls (72).

HLApers enables accurate quantification of HLA gene- and allele-specific expression from whole-transcriptome RNA-seq data (11). It provides both HLA genotyping and allele-specific expression quantification and has two pipelines options implemented; one for the STAR mapper combined with Salmon (73) for expression quantification and one for kallisto pseudoaligner (74) providing both the HLA genotyping and expression quantification. To reduce the possibility of multi-mapping reads in the expression quantification step, HLApers uses a personalized index, which comprises only the HLA allele sequences carried by the individual. The personalized pipeline of HLApers demonstrated several advantages such as higher accuracy in read alignment, HLA allele-specific expression quantification, and identification of causal eQTLs. A recent study applied HLApers to measure allele-level expression of HLA class I genes in unstimulated and stimulated PBMCs (30).

ArcasHLA, a python-based pipeline was initially developed for HLA genotyping from RNA-seq reads using kallisto, however, the authors later added the feature for allele-specific expression quantification currently allowing both highly accurate HLA typing and expression analysis (66). By using this novel pipeline, the authors analyzed the clinical significance of HLA class I LOH in multiple tumor types using public RNA-seq data (75).

scHLAcount enables HLA allele-specific expression quantification from single-cell RNA-seq (scRNA-seq) data (67). The pipeline builds a personalized reference based on prior HLA genotyping results and quantifies HLA expression at the allele-level using UMIs. In contrast to the tools for bulk RNA-seq data, scHLAcount provides an option to study HLA allele-specific expression and HLA loss of heterozygosity (LOH) at the single-cell resolution.

Although, these computational tools are advantageous by enabling mining of existing RNA-seq datasets, they face similar challenges as NGS-based HLA typing software with short RNA-seq reads multi-mapping to several alleles or even genes due to the high level of polymorphism and sequence similarity between HLA genes potentially resulting in biased expression estimates. ONT’s long-reads spanning over several exons might reduce ambiguous read alignment in expression quantification. However, currently, there is only one public computational tool, HLAXPress (9), in addition to Athlon2, for ONT long-read RNA-seq data, allowing HLA allele-specific expression quantification. The low throughput of ONT RNA-seq and low number of publicly available ONT RNA-seq datasets have potentially hindered the development of such tools (46). Additionally, although ONT’s whole transcriptome data has been sufficient for HLA gene-level expression analysis (10), accurate allele-level expression quantification may require an additional enrichment step to gain enough reads mapping to HLA. Moreover, in contrast to short-read technologies, ONT data has a higher error rate, which can hamper reliable UMI counting and accurate HLA genotyping. Lower number of reads and higher error rate are challenges that need to be considered in the development of novel computational tools for ONT RNA-seq. **Table 2** presents some examples of the criteria and methods in allele-specific expression quantification for different methodological approaches.

**Table 2:** Examples of different approaches in HLA allele-level expression quantification.

Approach	Requirements	Method in expression quantification	References
Illumina bulk RNA-seq and HLApers	Whole-transcriptome RNA-seq data (paired end reads in fastq format)	HLA genotyping is first done by aligning RNA-seq reads against all known HLA allele sequences. HLA expression is estimated by aligning RNA-seq reads against a personalized index containing references for individual-specific HLA alleles based on the genotyping results. HLA allele-specific expression is reported as the number of reads aligning to each allele.	(11)
Illumina bulk RNA-seq with UMIs and HLAXpress	5’end RNA-seq data with UMIs (paired end reads in fastq format) Prior HLA genotyping of samples for the personalized index	Expression levels are determined by first aligning RNA-seq reads against the reference sequences of known HLA alleles carried by an individual and then by counting the unique UMIs per each HLA allele.	(9)
ONT bulk RNA-seq and Athlon2	Amplicon-based RNA-seq data with UMIs tagged in the 5’end (ONT 1D reads in fastq format)	HLA genotyping is performed using the NGSengine bioinformatics pipeline and HLA allele-specific expression is quantified with Athlon2 pipeline by counting the UMI-tagged HLA-specific reads aligning to an allele.	(13)
Illumina scRNA-seq with UMIs and scHLAcount	Preferably 5’end RNA-seq data with UMIs (aligned reads in BAM format, cell barcodes) Prior HLA genotyping of samples	Prior knowledge from genotyping is utilized to construct a personalized reference. HLA allele-specific expression is determined by using pseudoalignment resulting in a matrix with allele-specific UMI counts.	(67)

UMI, unique molecular identifier; ONT, Oxford Nanopore Technologies.

## HLA expression in human diseases

Despite being crucial for protective immunity against various pathogens, the huge allelic variation of HLA is also responsible for autoimmune reactivity (35). In addition to allele polymorphisms as the susceptibility risk for autoimmune diseases, there is increasing evidence of associations between HLA expression and human diseases. However, in many cases further studies are needed to confirm whether the expression alone is the predisposing factor and how the regulatory variation affects differential expression in diseases. Elevated HLA expression levels have been associated with inflammatory bowel diseases (56, 76), scleroderma patients with interstitial lung disease (77), ankylosing spondylitis (78), Graves’ disease (79), systemic lupus erythematosus (80), rheumatoid arthritis (72), and multiple sclerosis (81). Additionally, in celiac disease and type 1 diabetes, higher expression of DQA1*05 and DQB1*02 alleles was found in patients when compared to healthy controls (82, 83). There is evidence that also low HLA expression predisposes to diseases. Decreased HLA expression levels have been associated with cystic fibrosis (84), immunoglobulin A nephropathy (85), end stage renal disease and acute allograft rejection (86). In addition to multiple autoimmune diseases, differential HLA expression has also been associated with infectious diseases i.e. high *HLA-C* expression in HIV viral control (42, 44, 56) and high *HLA-A* expression in higher HIV viremia (87). For class II genes, associations exist between high *HLA-DP* expression and in hepatitis B virus infection (54) and reduced expression of *HLA-DR* on monocytes and severity of COVID-19 disease (88). Strong HLA expression is an important factor for anti-tumor immunity, and thus downregulation or loss of HLA expression is a common immune escape mechanism in cancer (21). Indeed, several studies have reported downregulation of HLA expression in lung cancer (89, 90), gastric cancer (91), classic Hodgkin lymphoma (92), and in Merkel cell carcinoma (93). Due to the importance of HLA expression for immune response against tumor cells, high HLA expression has been associated with favorable outcomes and prolonged survival in several cancers (94–97). Opposed to this, high expression of non-classical HLA genes capable of suppressing immune responses are shown to correlate with poor prognosis (98, 99). In many disease studies, HLA expression has been determined from blood, however, to study the role of allele-specific expression in T cell maturation, the expression levels should be measured also from thymus samples. A weaker self-antigen presentation of low expression alleles could lead to impaired negative selection of T cell clones further resulting in elevated risks for the breakdown of tolerance and autoimmune diseases.

## Role of HLA allele-specific expression in HSCT

Although, HLA expression analysis is not considered in the current donor-recipient matching, there is evidence for the relevance of HLA allele-specific expression in HSCT. Several studies have associated differential HLA allele-specific expression with detrimental effects such as GvHD after the HSCT (40, 41, 100). Mismatched *HLA-C* alleles with high cell surface expression levels (mean fluorescence intensity) were identified as the key determinants for the increased risk for acute GvHD (aGvHD) and mortality indicating that high expression of patient’s allotypes enhances the graft-versus-host recognition (40). In the study of Petersdorf et al. (40) particularly the highly expressed C*14 allotype was associated with poor outcome after HSCT and was thus considered as a non-permissive mismatch. Another study also associated patient mismatched C*14:02 with high risk of severe aGvHD, but found no association between the HLA-C expression and the HSCT outcome although the HLA-C*14 allotype was expressed at the highest level (101). High allele expression predisposing to aGvHD is also found in *HLA-DPB1*. Two studies demonstrated that the risk of aGvHD was greater for patients with highly expressed *HLA-DPB1* alleles who received an *HLA-DPB1* mismatched transplant from a donor with low expression *HLA-DPB1* alleles (41, 100). Therefore, in case no matched donors are available, consideration of HLA expression could enhance the donor selection by helping to avoid mismatching against high-expression allele and thus lower the risks in HSCT. Additionally, identified low expression alleles as tolerated mismatches could broaden the donor pool. Interestingly, cell surface expression of *HLA-C* allotypes is consistent across populations denoting C*03 as low expression allele and C*14 as high expression allele (42). At the transcript level, there are similar findings for the expression levels of these alleles (8, 11, 30). However, there are also conflicting reports (9, 14) suggesting that allele-level expression is not necessarily universal and possibly population-dependent at least to some extent.

## Conclusions and perspectives

The advent of NGS technologies has revolutionized the study of HLA expression. It has led to the rapid development of RNA-seq based laboratory methods as well as computational tools for HLA expression quantification ready to be applied to existing datasets. As it can be challenging to obtain reliable results due to the high polymorphism and high sequence homology in HLA, many of the current methods rely on the use of a patient-specific personalized index in the expression quantification step. With continuous improvements in their sequencing accuracy, long-read NGS technologies such as ONT and PacBio could further increase the accuracy of HLA allele-specific expression quantification. Earlier HLA expression studies have mainly focused on quantifying HLA allele-specific expression from bulk RNA-seq data. However, particularly with class II genes, without information on the proportion of immune cells in the sample, it is hard to tell whether the expression level merely reflects the number of APCs in the sample. ScRNA-seq methods capable of distinguishing HLA expression from single cells enable more accurate comparisons between tissues and samples. Furthermore, spatial RNA-seq methods allow expression quantification between different cells in solid tissues (102). These methods would permit the study of loss of HLA expression in distinct spatial tumor clones. Determination of HLA expression is important in the selection of immune cell therapy. T-cell based therapies are dependent on strong HLA expression (103), whereas natural killer cell -based therapies rely on missing inhibitory ligands of killer-cell immunoglobulin-like receptors on the cell surface (104). Since, the previous studies have already demonstrated HLA allele-specific expression in healthy PBMCs, the focus in the future should be in investigating the role of HLA allele-specific expression in different tissues and diseases and how the expression changes in response to different stimuli and medications. Additionally, more information is needed on the dynamic changes in expression of HLA alleles over time from longitudinal samples from the same individual. Finally, the expression regulation of specific HLA alleles is still poorly understood. Therefore, by studying the methylation levels or using the expression quantitative loci (eQTL) analysis combining HLA expression together with data of non-coding variation obtained from genome-wide association studies might help to find potential factors affecting HLA allele-specific expression. By using scRNA-seq technology, the potential eQTLs behind differential expression could be identified even at the single-cell level.

## Author contributions

TJ, JP, and PS conceptualized the article. TJ wrote the first version of the manuscript. All the authors revised the final version of the manuscript and approved the submitted version.

## Funding

This work was financially supported by a grant from the Doctoral Programme in Biomedicine of the Doctoral School in Health Sciences, University of Helsinki. The funding body had no role in the study design or writing the manuscript.

## Conflict of interest

The authors declare that the research was conducted in the absence of any commercial or financial relationships that could be construed as a potential conflict of interest.

## Publisher’s note

All claims expressed in this article are solely those of the authors and do not necessarily represent those of their affiliated organizations, or those of the publisher, the editors and the reviewers. Any product that may be evaluated in this article, or claim that may be made by its manufacturer, is not guaranteed or endorsed by the publisher.
